# Resistance and Not Plant Fruit Traits Determine Root-Associated Bacterial Community Composition along a Domestication Gradient in Tomato

**DOI:** 10.3390/plants11010043

**Published:** 2021-12-23

**Authors:** Lisanne Smulders, Victoria Ferrero, Eduardo de la Peña, María J. Pozo, Juan Antonio Díaz Pendón, Emilio Benítez, Álvaro López-García

**Affiliations:** 1 Department Enviromental Protection, Estación Experimental del Zaidín (EEZ), CSIC, 18008 Granada, Spain; emilio.benitez@eez.csic.es; 2 Department of Biodiversity and Environmental Management, Campus de Vegazana s/n, University of León, 24071 León, Spain; vferrv@unileon.es; 3 Department of Plants and Crops, Faculty of Bioscience Engineering, Ghent University, Coupure Links 653, 9000 Ghent, Belgium; eduardo.deLaPena@ugent.be; 4 Department Soil Microbiology and Symbiotic Systems, Estación Experimental del Zaidín (EEZ), CSIC, 18008 Granada, Spain; mariajose.pozo@eez.csic.es (M.J.P.); alvaro.lopez@eez.csic.es (Á.L.-G.); 5 Finca Experimental “La Mayora” CSIC, Instituto de Hortofruticultura Subtropical y Mediterránea (IHSM-UMA-CSIC), 29750 Málaga, Spain; diazpendon@eelm.csic.es; 6 Department Animal Biology, Plant Biology and Ecology, Universidad de Jaén, 23071 Jaén, Spain; 7 Instituto Interuniversitario de Investigación del Sistema Tierra en Andalucía (IISTA), 18006 Granada, Spain

**Keywords:** breeding, microbiomes, rhizosphere bacterial communities, tomato domestications, traits

## Abstract

Soil bacterial communities are involved in multiple ecosystem services, key in determining plant productivity. Crop domestication and intensive agricultural practices often disrupt species interactions with unknown consequences for rhizosphere microbiomes. This study evaluates whether variation in plant traits along a domestication gradient determines the composition of root-associated bacterial communities; and whether these changes are related to targeted plant traits (e.g., fruit traits) or are side effects of less-often-targeted traits (e.g., resistance) during crop breeding. For this purpose, 18 tomato varieties (wild and modern species) differing in fruit and resistance traits were grown in a field experiment, and their root-associated bacterial communities were characterised. Root-associated bacterial community composition was influenced by plant resistance traits and genotype relatedness. When only considering domesticated tomatoes, the effect of resistance on bacterial OTU composition increases, while the effect due to phylogenetic relatedness decreases. Furthermore, bacterial diversity positively correlated with plant resistance traits. These results suggest that resistance traits not selected during domestication are related to the capacity of tomato varieties to associate with different bacterial groups. Taken together, these results evidence the relationship between plant traits and bacterial communities, pointing out the potential of breeding to affect plant microbiomes.

## 1. Introduction

Over time, agricultural techniques have changed to meet the growing demand for food and agricultural products of the world population. Agricultural productivity has increased over the last century through, amongst others, improved varieties and increased use of agrochemicals, leading to environmental issues, weakened cropping systems and increased demands for sustainable agriculture [1].

The plant rhizosphere contains a large diversity of microorganisms that are involved in multiple ecosystem services, including an increase in plant nutrition and disease suppression [2]. The composition of the plant-associated microbiome is determined by the interplay of the host plant characteristics and the surrounding soil conditions [2,3,4]. Plants structure their microbiome through the release of a specific blend of exudates, the composition of which depends on the plant species or variety, being phylogenetically conserved, even at the genotype level [5,6]. Conversely, soil microorganisms affect a variety of host plant traits, including nutritional content and morphology, as well as activating defence pathways and the emission of plant volatile organic compounds [2,5,6], thereby altering the interactions with insect herbivores or plague enemies aboveground [7].

Through domestication, modern plant varieties have been developed with different fruit shapes, colours and sizes adapted to consumer preferences [8,9]. Indeed, in many crops, breeding has focused on phenotype, such as fruit size and yield [9,10], and other traits favouring resistance and defence have been left aside, yielding crops more susceptible to pests and diseases [11,12,13]. As an example, between the studied traits that could impact plant resistance, glandular trichomes of tomato cultivars have been seen to provide refuges for pests that hamper host finding by parasitoids [14]. Moreover, reduced volatile emissions due to domestication have been shown in maize, cranberry and lupin [15,16,17], with potential effects on pest repulsion/attraction. In a previous field study evaluating the response of 23 tomato accessions to important agricultural pests, higher tolerance levels were evidenced in wild and early domesticated accessions than in modern ones, although the differences could not be linked to the phylogenetic distance between accessions [11]. The effects of domestication on plant traits are, thus, complex, and a diversity of genes and gene sets have been associated with domestication (reviewed in [18]). The reduced genetic and associated trait diversity in domesticated varieties could indirectly impact ecosystem services, such as pest control by natural enemies [14,19,20]. Indeed, genetically diverse fields harbour diverse insect populations, including natural enemies, thereby improving biological pest control [19,20].

Despite this knowledge on domestication effects, the potential role of plant-associated microbiomes in plant resistance or tolerance to diseases has been overlooked. It is known that in many crops such as tomato, domestication and breeding together with increased use of pesticides and fertilisers have resulted in varieties with reduced investment in costly beneficial plant–microbe interactions below and aboveground [1,6,7,9,14,21]. Genetic variation between varieties affects morphological traits such as root growth, architecture and exudate composition, which may impact microbiome assembly [22]. It is then assumed that, despite the lack of studies on plant-associated microbiomes along the domestication process, humans have potentially altered microbiome compositions by, e.g., altering plant metabolic activity [12,23,24].

There are potential links between plant fruit traits (selected during breeding) and the degree of domestication with associated soil microbiomes. Studies in pear and pepper have shown that varieties with large fruits or high fruit sets divert more photosynthates to growth than smaller fruited varieties [25,26], potentially altering the release of exudates through roots. Modern cultivars also differ in root architecture compared to their wild relatives, and the consequent changes in root exudation profiles may also impact rhizosphere community composition [6]. In general, modern cultivars have shallower roots due to readily available macronutrients and water in agricultural fields and higher exudation of simple sugars [27]. A study comparing wild and modern bean varieties showed that differences in root length partly explains the divergence in rhizobacterial communities [28]. Since resistance traits are not very specific and often involve changes in metabolite profiles at the root or systemic level, we expect that soil-associated bacterial communities are affected to a wider extent by these traits than those related to fruit. Furthermore, resistant plants are expected to recruit microbes to alleviate stress [22].

Previously, we explored how domestication impacted a selection of 27 tomato accessions from plant ancestors to modern cultivars in terms of function, revealing maintenance of all functional characteristics, with some specific pathways being modified [29]. In this study, we extend on these results using the data of 18 of these tomato varieties to (i) reveal trends in tomato traits during domestication by differentiating between fruit and resistance traits, and (ii) explore to what extent these traits are associated with root-associated bacterial community composition. We expect that crop domestication caused differences in resistance and fruit traits across tomato varieties, with resistance traits having a higher impact on bacterial community composition. Knowledge on the extent to which root-associated bacterial microbiomes covary with targeted (fruit) and untargeted (resistance) domestication traits will provide insights into the potential consequences of breeding for plant microbiome composition.

## 2. Results

### 2.1. Plant Traits Affected by Domestication

**Figure 1:**
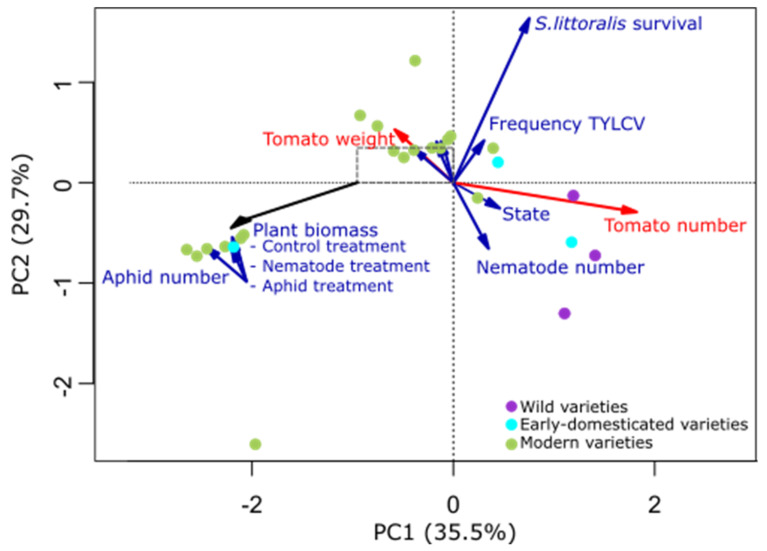
Principal components analysis (PCA) of plant traits (fruit in red and resistance in blue) of 18 varieties of tomato (*Solanum lycopersicum* Mill., *S. habrochaites* and *S. pimpinellifolium*). Tomato varieties were classified into wild (purple), early domesticated (light blue) and modern (green): TYLCV, tomato yellow leaf curl virus frequency of infection; *S. littoralis*, *Spodoptera littoralis*; State, plant state according to symptom severity scale. A detail of shorter axes is provided (dashed square) to improve readability. For clarification, very short axes, i.e., those of minor importance, were not shown in the ordination.

The PCA ordination using plant traits from both experiments showed a clear trend from modern to wild tomato varieties from left-up to right-down positions of the ordination (Figure 1); wild tomato varieties produced more and smaller tomatoes than early domesticated and modern varieties, whereas modern varieties produced the highest plant biomass (both with and without pests) (see Table S1 for details on traits).

### 2.2. Plant Domestication Influence Soil Bacterial Communities

Some effect of domestication was observed on the bacterial community diversity, which was described in a previous paper [29]. Shortly, minority phyla such as Acidobacteria and Gemmatimonades were less present in wild tomato species, as in modern tomato. At the family level, wild tomato species had a lower abundance of Gemmatimonadaceae, Microbacteriaceae and Streptomycetaceae and a higher abundance of Sphingomonadaceae compared with modern tomato. Minor changes were observed at the OTU level, with reduced evenness in wild tomato.

### 2.3. Plant Traits and Soil Characteristics Influence Soil Bacterial Communities

**Figure 2:**
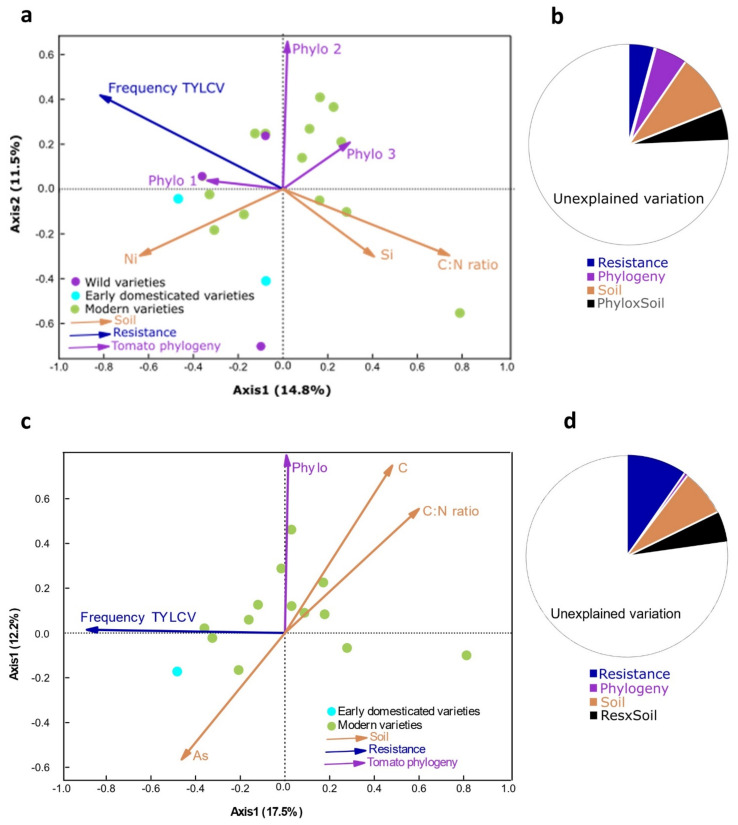
Redundancy analysis of bacterial OTU composition of tomato driven by plant (resistance and fruit morphology), soil (nutrients) and tomato phylogeny: (**a**) redundancy analysis; (**b**) varpart, including all three groups of tomato varieties; (**c**) redundancy analysis; (**d**) varpart, excluding wild varieties.

Bacterial OTU richness and Simpson and Shannon diversity indices significantly increased with *S. littoralis* survival and occurrence of tomato yellow leaf curl virus (TYLCV), mainly due to resistance traits showed by the variety Periana, and decreased with soil Si and Sr (Table S2). A set of plant traits and soil characteristics was found to drive the OTU composition of bacterial communities; in other words, they represented the minimum number of variables that explained a major proportion of OTU variation across samples [30]. For resistance, TYLCV was the most significant factor explaining the variation in soil bacterial OTU communities (Table S3). CN ratio, Si and Ni in soil were similarly selected. No significant effects were identified for fruit traits. These traits, together with the tomato phylogeny (four PCOA axes), were used to partition the variation of OTU bacterial community composition (Figure 2a,b). Total explained variation reached 25% (Table S4). Soil (9.4%) explained most variation (alone or combined with tomato phylogeny), whereas tomato phylogeny (5.4%) and resistance (4.3%) explained a smaller part of the variation.

The variation partitioning was repeated, excluding wild varieties, as a way to avoid bias caused by the fact that cultivated tomato belonged to the same tomato species (*S. lycopersicum*). Conversely, wild tomatoes belonged to different plant species. In this analysis, TYLCV was again selected together with soil CN ratio, C and As. In this case, the total explained variation reached 23% (Figure 2c,d), with resistance explaining most variation (9.7%), followed by soil (7.4%) and phylogeny (1%).

## 3. Discussion

In this study, the effect of tomato domestication on root-associated bacterial community composition was observed from a trait perspective, building upon results described in our previous study on functional diversity [29]. This second approach allows for a mechanistic interpretation of the identified patterns. Fruit traits (i.e., tomato number and weight) varied according to tomato domestication. However, resistance traits, non-related to tomato domestication, drove most of the explained variation in bacterial OTU composition.

The spectra of studied tomato varieties showed that modern varieties contained heavier and fewer fruits than wild or early domesticated varieties, consistent with modern tomatoes being large and diverse in shape, whereas wild tomatoes are generally small and round [31]. Even though tomatoes have not been selected for their root-associated microbiome, their structure might be altered through changes in root exudates, usually tied to root morphological characteristics, which feed and filter root microbiota [6,22,32,33].

Since domestication has often been accompanied by creating crops with shallow roots, shifts in traits such as leaf size and root architecture, resulting in increased litter quality and a lower C:N ratio, could influence microbial community composition [34]. Furthermore, Leff et al. found that faster-growing sunflower varieties had lower bacterial diversity in the rhizosphere [24]. Moreover, some authors linked bacterial-associated diversities to increased plant growth [35]. However, we did not find an effect of aboveground plant morphology on root-associated bacterial community structure. This could be due to the lack of links between the morphological traits used in this study and the more impacting belowground traits on root-associated microbiomes. For example, Legay et al. showed that belowground (root-associated) traits, such as root C:N ratio and root diameter, explained more variation in microbial properties than aboveground (leaf) traits [36]. Furthermore, the main driver of bacterial communities close to the roots is recruitment from the bulk soil. However, even though some authors found differences in microbial communities between bulk and rhizosphere soil [37], others found a difference in both compartments with the phyllosphere [38]. In addition, diversity indices were found to be lower or higher in the rhizosphere compared with bulk soil [39,40]. The effect of soil nutrients suggests that environmental variables such as soil type impact microbial communities [2,33]. For example, Peiffer et al. showed in different maize varieties that plant genotype affects OTU richness within a field, and this genotypic effect varies between field environments [41]. Thus, it was suggested that environmental factors such as pH and geographic patterns interact to shape maize rhizosphere microbiota. Furthermore, microbes themselves influence community structure by producing secondary metabolites such as antibiotics and toxins to compete with other microbes and successfully establish in the rhizosphere [2,5].

Despite this evidence, explaining the change in bacterial microbiomes through domestication seems difficult due to the contradictory results often found. For example, Leff et al. observed little effect of the domestication of sunflower on overall rhizosphere bacterial communities [24]. By contrast, Shenton et al. found that rhizosphere bacterial communities of wild rice differ in species richness and composition compared with cultivated rice, which was not correlated to the genetic distance of the plants [42]. However, in our study, resistance traits that were not aligned with the domestication degree (see [11]) were responsible for an important fraction of the variation on root-associated bacterial communities, especially between domesticated varieties, aligning with the evidence that plants shape microbial communities as an additional layer of defence. For example, plants under attack may recruit microorganisms that alleviate biotic stress or actively repress pathogen proliferation [22,43]. As far as we know, only one study has observed a difference between microbial communities associated with wild and domesticated plants, in this case, rice, in their response to a biotic challenge [44]. Plant resistance may also impact the functional profile of associated bacterial communities. For example, common bean genotypes resistant to *Fusarium oxysporum* contained bacterial communities enriched in genes encoding antifungal compounds [45]. Plant genetic factors related to immunity were shown to play a role in structuring the microbial community [46]. For example, Lebeis et al. showed how plant defence hormones, especially salicylic acid, shape root bacterial communities [47]. We also found a positive correlation between bacterial diversity and reduced plant resistance traits (*S. littoralis* survival and TYLCV infection). In this sense, it has been proposed that higher bacterial diversities should be associated with increased plant resistance due to the observed recruitment of beneficial microbes [9,48,49]. However, our results clearly aligned with Doornbos et al., who postulated that more susceptible plants harbour more diverse bacterial communities than resistant plants [43], but this is not always the case [23,24].

The limitations of sequencing technology were described in a recent review [50]. Most studies focus on bacteria, while other organisms such as fungi, viruses and archaea may be important as well [24,38,39]. Furthermore, often studies are conducted on a small scale, which limits detection of low-abundance taxa that could have a leading role in microbial community structure and function. DNA-based sequencing does not allow to determine whether the bacterial OTUS are functional. However, it is possible to study potential functions, which we found to be affected by domestication [29]. Furthermore, the diversity profile found could depend on the primer set used [41]. The V3–V4 region, as used in this study, was found to detect the highest phylum diversity compared with other 16S primers.

## 4. Materials and Methods

### 4.1. Field Experiment

We used 27 tomato accessions from a field experiment described previously [29]. Briefly, the field experiment was performed as follows. Seeds of 27 *Solanum lycopersicum* Mill., *S. habrochaites* and *S. pimpinellifolium* accessions were obtained from the Instituto de Hortofruticultura Subtropical y Mediterránea “La Mayora” (IHSM-UMA-CSIC) germplasm bank, based on their degree of domestication (Table S1A). On 19 April 2018, 10 one-month-old seedlings per variety, 270 in total, were randomly distributed in an experimental field in the IHSM La Mayora (Málaga, Spain, 36.77° N, 4.04° W). The field soil is classified as a eutric regosol soil [51]. Plants were exposed to the natural community of insects and became naturally infected by two common viral diseases transmitted by whiteflies: tomato yellow leaf curl virus (TYLCV) and tomato chlorosis virus (ToCV). Plants were grown until 16 July 2018, when they were in the fruiting stage.

At harvest, the soil attached to the main and secondary roots was taken by shaking. The root-associated soil from each plant was placed in separate plastic bags and kept at 4 °C until laboratory analyses (60 days). Then, samples from each variety were pooled and ground together using a mortar and pestle and sieved twice (2-mm mesh) and immediately stored at −20 °C until molecular analyses were performed. The aboveground biomass was weighted, the number of tomatoes produced counted and the total tomato fruit weight measured (Table S1B). Freezing is often considered the best option to store soil samples for microbiome analysis. However, research shows that storage at 4 °C for up to 30 days has a minimal effect on microbiome composition [52,53,54]. We used data from 18 tomato accessions that were selected because there were greenhouse data on resistance traits available (see Section 4.2).

On 28 June 2018, plant state was scored according to the symptom severity scale described by Kone et al. [55], which runs from 0 = no disease symptoms to 10 = severe leaf distortion/necrosis/narrowed or shoes-string leaf (Table S1B).

The frequency of virus infection per tomato variety was performed using tissue blot hybridisation methodologies described by Navas-Castillo et al. and Fortes et al. [56,57] for TYLCV and ToCV, respectively. Virus frequency and plant state were used as resistance traits of each tomato variety (Table S1B). Table S1 shows the average values for each tomato variety.

### 4.2. Resistance to Pests Data

In a previous glasshouse experiment performed by Ferrero et al. (see details in [11]) in steam-sterilised sand–peat mixture, the response of the same 18 tomato varieties to root-knot nematodes, aphids and *Spodoptera littoralis* were determined after 6 weeks of growth. Plant biomass (dry weight) was measured under control (no pest) and pest treatment conditions.

For aphids, the total number of individuals were counted; for nematodes, the number of root knots/mg root (nematode number); and the mean increase in *S. littoralis* larvae weight per day (survival) was determined. These were included in the list of plant resistance traits (Table S1D). The averages across the tomato variety replicates were used as the trait values characteristic of each variety.

The plant phylogenetic tree developed by Ferrero et al. [11] was used to extract a phylogenetic distance matrix between tomato varieties. The variation in this matrix was decomposed by principal coordinates analysis, and the four generated axes were fed into subsequent analyses (capscale function in R package Vegan).

Tomato varieties were grouped depending on their degree of domestication into wild (*Solanum habrochaites* and *S. pimpinellifolium*) and early domesticated and modern (including *S. lycopersicum* var. *cerasiforme* and *S. lycopersicum*) (Table S1A). This classification was established by Ferrero et al. [11] and here verified using a k-means clustering analysis based on the measured traits [58].

### 4.3. Soil Chemical Characterisation

For each variety, two replicates of air-dried field soil samples were used to determine chemical properties at the Scientific Instrumentation Service, EEZ-CSIC, Granada, Spain (Table S1C). Total N and soil organic C were determined with the aid of the Leco-TruSpec CN elemental analyser (LECO Corp., St Joseph, MI, USA). Total mineral content was determined by the digestion method with HNO_3_ 65%:HCl 35% (1:3; v-v) followed by analysis using inductively coupled plasma optical emission spectrometry (ICP-OES) (ICP 720-ES, Agilent, Santa Clara, CA, USA).

### 4.4. Molecular Analyses of Soil Bacteria

DNA was extracted separately from eight 1 g soil subsamples using the bead-beating method with the PowerSoil^®^ DNA Isolation Kit (MoBio Laboratories, Solana Beach, CA, USA) according to the manufacturer’s instructions. Extracts of four subsamples were pooled and further concentrated at 35 °C to a final volume of 20 μL using a Savant Speedvac^®^ concentrator, resulting in two replicates per variety. Bacterial communities were analysed using Illumina MiSeq, and to determine the bacterial communities, we amplified the V3–V4 hypervariable regions of the 16S rRNA gene using the ProV3V4 primers with the following sequences: 5′ CCTACGGGNBGCASCAG 3′ and 5′ GACTACNVGGGTATCTAATCC 3′ [59,60]. The amplified region was approximately 464 bp. The products were sequenced on the Illumina MiSeq platform using a 2 × 250 nucleotide paired-end protocol (genomic facilities of the López-Neyra Institute of Parasitology and Biomedicine, IPBLN-CSIC, Granada, Spain). To minimise amplification of mitochondria and chloroplasts, blockers were used [60]. Between the two PCR steps, amplicons were purified and after the second PCR step, amplicons were pooled in an equimolar manner.

SEED2 was used for the initial steps of processing the resulting sequences [61]. First, we merged forward and reversed sequences. Then, sequences containing ambiguous bases (N) and with a quality score below 30 were removed. Primer sequences were removed and sequences trimmed to 400 bp. Afterwards, the sequences were clustered into operational taxonomic units (OTUs) using the UPARSE method by setting the OTU radius to 3%, so selecting sequences at 97% similarity. OTUs with just one read were removed together with chimeric sequences. Finally, a consensus sample x OTU matrix was prepared and the most abundant sequence per OTU was selected as representative. Taxonomy was assigned to each OTU using the classify.seqs algorithm in mothur software together with the SILVA database version 132 [62,63]. At this stage, no archaea were detected in the samples. The rarefaction curves were visualised in Microbiome Analyst to confirm that all samples reached the plateau [64,65] (Figure S1). The two replicates per variety were summed for the statistical analysis.

All Illumina sequence raw data were deposited in the Sequence Read Archive (SRA) service of the European Bioinformatics Institute (EBI) database (BioProject ID: PRJNA693664).

### 4.5. Statistical Analyses

Before the statistical analyses, plant traits were averaged per variety and log transformed. The OTU abundance table was Hellinger transformed. The relationship between fruit and resistance traits and the domestication degree of the tomato varieties was visualised via principal components analysis.

Bacterial OTU richness and Simpson and Shannon diversity indices were calculated using the abundance OTU x sample matrix after the Hellinger transformation of data. Their relationship with plant traits and soil variables were tested via Spearman correlation.

The relative influence of tomato fruit traits, resistance traits, phylogeny of tomato and soil chemical composition on bacterial OTU composition was tested by variation partitioning approach based on redundancy analysis (RDA, 29). The minimum number of variables inside each of these explaining factor classes explaining a major part of OTU variation was selected via forward selection using ordistep.

These analyses were carried out in R software using the R packages Vegan [66], picante [67], FD [68], dplyr [69] and lme4 [70]. The R script is available as Supplementary Materials.

## 5. Conclusions

In our study, we found that fruit traits (tomato number and weight) varied according to tomato domestication, while resistance traits drove most of the explained variation in bacterial OTU composition. These mechanisms highlight a direct effect of plant defence mechanisms on root-associated bacterial community composition and are consistent with the found dependence of bacterial communities on resistance traits. In summary, this study reveals how non-targeted traits during domestication shape the bacterial community of tomato, but further research is required to confirm the mechanisms behind the relationship between bacterial communities and plant resistance.

## Data Availability

All Illumina sequence raw data were deposited in the Sequence Read Archive (SRA) service of the European Bioinformatics Institute (EBI) database (BioProject ID: PRJNA693664).

## Supplementary Materials

The following are available online at https://www.mdpi.com/article/10.3390/plants11010043/s1, Figure S1: Rarefaction curves of 18 varieties of tomato (*Solanum lycopersicum* Mill. *S. habrochaites* and *S. pimpinellifolium*). Tomato varieties were classified into wild (purple). Early domesticated (light blue) and modern (red). Table S1: Experimental variables included in the multivariate analysis: (A) domestication degree per tomato variety; (B) data from the field experiment: plant traits; (C) data from field experiment: soil characterisation; (D) data from Ferrero et al. 2019: plant traits. Table S2: Pearson correlation test of bacterial diversity indexes and plant traits and soil variables. R coefficients are shown. Asterisks indicate significance: * *p* < 0.05; ** *p* < 0.01. For details on plant traits, see Table S1. Table S3: Stepwise model selection of redundancy analyses for plant traits (fruit and resistance) and soil nutrient variables. Asterisks indicate significance: *p* < 0.1; * *p* < 0.05; ** *p* < 0.01. For details on plant traits, see Table S1. Table S4: Variation partitioning of bacterial OTU community composition in plant traits (fruit and resistance), tomato phylogeny and soil variables, either considering the whole dataset or only domesticated tomato varieties. Asterisk indicates significant *p* values: *p* < 0.1; * *p* < 0.05. For details on plant traits, see Table S1. R script and OTU Table are also available as Supplementary Files. Raw data on plant traits are available upon request.
