# Supplementary material for: Resistance and Not Plant Fruit Traits Determine Root-Associated Bacterial Community Composition along a Domestication Gradient in Tomato
Source: Plants (Basel). 2021 Dec 23;11(1):43. doi: 10.3390/plants11010043 (PMC8747438; doi:10.3390/plants11010043)
Supplement: Supplementary file 1 [file plants-11-00043-s001.zip › SupplementaryTables.docx]

Supplementary Material

**Table S1.** Experimental variables included in the multivariate analysis. A) Domestication degree per tomato variety. B) Data from the field experiment: plant traits. C) Data from field experiment: soil characterization. D) Data from Ferrero et al. 2019: plant traits.

| **Domestication degree** | | |
| --- | --- | --- |
| **Tomato variety** | **Species** | **Domestication degree** |
| H. de Toro | *Solanum lycopersicum* | Modern |
| BC5 | *Solanum lycopersicum* | Modern |
| Edkawi | *Solanum lycopersicum* | Modern |
| Flor Baladre | *Solanum lycopersicum* | Modern |
| Kalohi | *Solanum lycopersicum* | Modern |
| LA1589 | *Solanum pimpinellifolium* | Wild |
| Marmande | *Solanum lycopersicum* | Modern |
| Melillero | *Solanum lycopersicum* | Modern |
| Mex 89 | *Solanum lycopersicum* var*. cerasiforme* | Early-domesticated |
| Moneymaker | *Solanum lycopersicum* | Modern |
| Monita | *Solanum lycopersicum* | Modern |
| Moruno | *Solanum lycopersicum* | Modern |
| PE55 | *Solanum lycopersicum* | Early-domesticated |
| De Penjar | *Solanum lycopersicum* | Modern |
| Periana | *Solanum lycopersicum var. cerasiforme* | Modern |
| PI134418 | *Solanum habrochaites* | Wild |
| San Marzano | *Solanum lycopersicum* | Modern |
| T0 93715 | *Solanum pimpinellifolium* | Wild |

| **B) Plant traits field experiment.** Plant state: Plant symptom characterization; Frequency ToCV: Percentage of tomato replicates with chlorosis virus detected with tissue-blot hybridization; Frequency TYLCV: Percentage of tomato replicates with yellow leaf curl virus detected with tissue-blot hybridization. | | | | | | | |
| --- | --- | --- | --- | --- | --- | --- | --- |
| **Tomato variety** |  | **Total plant biomass (Kg)** | **Tomato fruit weight (Kg)** | **Tomato fruit number** | **State** | **Frequency ToCV** | **Frequency TYLCV** |
| H. de Toro |  | 0.41 | 99.2 | 0.5 | 3.00 | 1.00 | 0.8 |
| BC5 |  | 0.57 | 207.91 | 5.7 | 4.3 | 0.20 | 0.3 |
| Edkawi |  | 0.325 | 107.05 | 0.875 | 2.75 | 1.00 | 0.7 |
| Flor Baladre |  | 0.33 | 225.04 | 0.33 | 2.22 | 1.00 | 0.89 |
| Kalohi |  | 0.39 | 165.33 | 1.1 | 2.2 | 1.00 | 0.6 |
| LA1589 |  | 0.526 | 51.06 | 61.3 | 6.6 | 0.80 | 0.8 |
| Marmande |  | 0.33 | 412.95 | 3.5 | 2.33 | 1.00 | 0.5 |
| Melillero |  | 0.52 | 201.94 | 1.8 | 2.7 | 0.9 | 0.4 |
| Mex 89 |  | 0.76 | 85.19 | 65.78 | 5.78 | 0.9 | 0.8 |
| Moneymaker |  | 0.363 | 403.82 | 4.2 | 3.00 | 1.00 | 0.7 |
| Monita |  | 0.29 | 98.24 | 2.09 | 2.45 | 1.00 | 0.7 |
| Moruno |  | 0.24 | 137.82 | 4.00 | 2.29 | 1.00 | 0.25 |
| PE55 |  | 0.28 | 46.00 | 5.17 | 3.00 | 1.00 | 0.625 |
| De Penjar |  | 0.6725 | 282.56 | 12.5 | 3.9 | 1.00 | 0.4 |
| Periana |  | 0.5 | 69.34 | 0.3 | 2.7 | 0.9 | 0.1 |
| PI134418 |  | 0.39 | 21.12 | 17.89 | 7.44 | 0.67 | 0.5 |
| San Marzano |  | 0.63 | 110.17 | 2.11 | 2.44 | 1.00 | 0.5 |
| T0 93715 |  | 0.63 | 7.88 | 15.2 | 7.4 | 0.4 | 0.6 |

| **C) Field experiment Chemical composition of soil (ppm (mg/Kg))** | | | | | | | | | |  |  |  |  |
| --- | --- | --- | --- | --- | --- | --- | --- | --- | --- | --- | --- | --- | --- |
| **Tomato variety** | **Nitrogen (%)** | **Carbon (%)** | **C:N ratio** | **Al** | **As** | **Ca** | **Cd** | **Co** | **Cr** | **Cu** | **Fe** | **K** | **Li** |
| H. de Toro | 0.29 | 5.48 | 19.07 | 32272.45 | 30.64 | 9512.67 | 2.29 | 21.63 | 47.01 | 39.16 | 32775.74 | 7891.33 | 29.24 |
| BC5 | 0.30 | 5.77 | 19.25 | 29903.35 | 26.45 | 10998.40 | 2.23 | 19.42 | 44.87 | 40.29 | 31155.15 | 7694.72 | 26.80 |
| Edkawi | 0.31 | 6.03 | 19.47 | 30849.13 | 29.29 | 9355.71 | 2.23 | 21.20 | 45.56 | 39.34 | 33169.75 | 7519.68 | 28.77 |
| Flor Baladre | 0.25 | 4.00 | 16.03 | 40469.74 | 30.25 | 8871.26 | 2.40 | 22.48 | 54.91 | 40.06 | 35588.47 | 9655.52 | 33.43 |
| Kalohi | 0.31 | 5.25 | 17.18 | 34563.67 | 27.25 | 11063.83 | 2.07 | 19.38 | 48.22 | 38.63 | 31031.98 | 8877.17 | 27.95 |
| LA1589 | 0.30 | 5.79 | 19.14 | 44769.84 | 32.79 | 7804.21 | 2.22 | 21.64 | 57.71 | 40.92 | 33620.68 | 11887.42 | 31.11 |
| Marmande | 0.36 | 9.51 | 26.35 | 47279.17 | 33.44 | 10332.83 | 2.09 | 22.12 | 59.18 | 38.17 | 31873.98 | 12308.45 | 28.36 |
| Melillero | 0.27 | 5.05 | 19.05 | 48191.47 | 32.23 | 8737.64 | 2.27 | 21.26 | 61.57 | 38.24 | 34490.29 | 12655.27 | 31.47 |
| Mex 89 | 0.24 | 3.57 | 14.78 | 36127.94 | 35.02 | 7748.17 | 2.40 | 22.77 | 50.77 | 40.46 | 35408.39 | 8917.46 | 30.71 |
| Moneymaker | 0.32 | 6.79 | 21.11 | 39290.18 | 31.92 | 8788.23 | 2.22 | 20.71 | 52.42 | 41.88 | 33572.50 | 9825.97 | 28.13 |
| Monita | 0.35 | 7.83 | 22.27 | 36863.42 | 27.85 | 9099.69 | 2.15 | 20.85 | 50.65 | 39.99 | 32321.24 | 9701.17 | 27.78 |
| Moruno | 0.31 | 6.69 | 21.30 | 33901.28 | 28.67 | 10937.73 | 2.13 | 20.73 | 47.21 | 38.34 | 31601.04 | 8521.75 | 26.27 |
| PE55 | 0.21 | 3.42 | 16.68 | 44845.01 | 37.77 | 8121.07 | 2.40 | 23.68 | 57.48 | 39.02 | 35385.81 | 11384.10 | 30.59 |
| De Penjar | 0.26 | 4.19 | 15.89 | 43683.51 | 31.67 | 8550.81 | 2.29 | 22.27 | 56.89 | 38.22 | 34266.37 | 11362.56 | 30.16 |
| Periana | 0.32 | 6.75 | 21.14 | 46608.99 | 23.73 | 11773.04 | 2.03 | 20.42 | 58.46 | 38.14 | 30811.28 | 12562.43 | 28.25 |
| PI134418 | 0.29 | 5.16 | 18.05 | 52079.30 | 35.37 | 8814.62 | 2.42 | 24.27 | 64.50 | 40.21 | 35617.88 | 13259.06 | 32.10 |
| San Marzano | 0.29 | 5.32 | 18.33 | 48274.30 | 25.99 | 7567.39 | 2.14 | 22.05 | 59.35 | 34.75 | 33049.06 | 12385.03 | 28.82 |
| T0 93715 | 0.33 | 5.44 | 16.44 | 34162.53 | 33.24 | 7739.21 | 2.26 | 21.82 | 48.03 | 38.65 | 33498.50 | 8794.56 | 27.40 |

| **Tomato variety** | **Mg** | **Mn** | **Na** | **Ni** | **P** | **Pb** | **S** | **Si** | **Sr** | **Ti** | **V** | **Zn** |
| --- | --- | --- | --- | --- | --- | --- | --- | --- | --- | --- | --- | --- |
| H. de Toro | 7987.08 | 846.29 | 0.04 | 45.93 | 707.46 | 22.47 | 411.73 | 2877.92 | 47.73 | 1148.59 | 59.06 | 125.51 |
| BC5 | 8344.68 | 771.62 | 0.03 | 40.74 | 715.53 | 21.64 | 489.64 | 2728.97 | 57.07 | 1128.88 | 56.16 | 131.67 |
| Edkawi | 7550.65 | 875.68 | 0.03 | 43.74 | 818.07 | 22.09 | 470.31 | 2652.96 | 58.89 | 1077.34 | 56.82 | 106.75 |
| Flor Baladre | 8372.00 | 863.50 | 0.04 | 48.04 | 767.09 | 23.16 | 373.89 | 3398.72 | 52.20 | 1318.87 | 69.95 | 109.48 |
| Kalohi | 8465.32 | 859.65 | 0.04 | 41.15 | 832.00 | 21.53 | 481.18 | 3204.44 | 54.61 | 1212.39 | 60.98 | 99.80 |
| LA1589 | 7584.11 | 888.58 | 0.05 | 45.09 | 968.39 | 23.32 | 409.23 | 4196.16 | 50.89 | 1375.55 | 73.44 | 84.85 |
| Marmande | 8032.74 | 925.72 | 0.06 | 43.99 | 992.85 | 23.92 | 487.64 | 4799.41 | 61.69 | 1490.84 | 77.27 | 84.34 |
| Melillero | 8438.64 | 807.50 | 0.05 | 45.56 | 887.12 | 23.33 | 388.67 | 4237.66 | 51.75 | 1534.15 | 79.85 | 83.94 |
| Mex 89 | 7884.02 | 980.78 | 0.03 | 48.31 | 1020.34 | 24.51 | 341.58 | 3054.83 | 42.80 | 1191.39 | 64.45 | 97.92 |
| Moneymaker | 8131.44 | 727.50 | 0.04 | 43.16 | 822.85 | 23.24 | 406.64 | 3350.30 | 54.84 | 1232.17 | 66.60 | 91.76 |
| Monita | 7800.04 | 793.15 | 0.04 | 43.02 | 1072.14 | 22.11 | 471.51 | 3359.55 | 53.64 | 1332.99 | 63.90 | 85.51 |
| Moruno | 8424.78 | 882.49 | 0.04 | 42.95 | 933.49 | 23.01 | 422.84 | 3230.94 | 56.79 | 1149.81 | 59.14 | 96.92 |
| PE55 | 7764.41 | 1022.06 | 0.05 | 48.33 | 1236.94 | 25.20 | 314.81 | 3843.06 | 47.52 | 1304.76 | 73.95 | 100.50 |
| De Penjar | 8187.77 | 874.02 | 0.05 | 45.86 | 1064.76 | 24.39 | 390.81 | 3542.49 | 50.39 | 1380.06 | 73.00 | 103.77 |
| Periana | 8992.66 | 729.31 | 0.06 | 40.29 | 937.59 | 22.04 | 547.08 | 4490.26 | 65.13 | 1555.12 | 76.24 | 96.26 |
| PI134418 | 8677.31 | 968.29 | 0.06 | 49.26 | 1174.52 | 24.65 | 414.23 | 3914.28 | 47.82 | 1479.69 | 84.41 | 93.77 |
| San Marzano | 7979.41 | 1177.24 | 0.06 | 49.03 | 992.56 | 21.64 | 347.10 | 4147.62 | 49.36 | 1422.21 | 79.08 | 84.83 |
| T0 93715 | 7232.60 | 955.40 | 0.03 | 45.16 | 1129.40 | 24.36 | 404.48 | 4089.26 | 47.52 | 1175.80 | 60.73 | 107.31 |

|  |  |  |  |  |  |  |
| --- | --- | --- | --- | --- | --- | --- |
| **D) Ferrero et al. (2019) resistance traits.** Averages per tomato variety. Total plant biomass (g): Total plant biomass (dry weight) of control plants (no pest attack); Spodoptera exigua survival: Mean increase in weight per day; Plant biomass (aphid treatment) (g): Plant biomass (dry weight) under aphid pressure; Aphid number: Number of aphids in the plant at the end of experiment; Plant biomass (nematode treatment)(g): Plant biomass (dry weight) under nematode pressure; Nematode number: Number of root knots/ mg root. | | | | | | |
| **Tomato variety** | **Total plant biomass (g)** | **Spodoptera exigua survival** | **Plant biomass (aphid treatment) (g)** | **Aphid number** | **Plant biomass (nematode treatment) (g)** | **Nematode number** |
| H. de Toro | 3.997 | 0.47 | 3.66 | 70.64 | 4.96 | 13.63 |
| BC5 | 3.708 | 0.33 | 3.13 | 32.86 | 3.42 | 47.14 |
| Edkawi | 4.746 | 0.60 | 2.56 | 66.83 | 4.01 | 79.41 |
| Flor Baladre | 3.818 | 0.60 | 3.09 | 67.60 | 4.10 | 27.21 |
| Kalohi | 3.901 | 0.33 | 3.30 | 81.80 | 4.21 | 21.23 |
| LA1589 | 2.815 | 0.27 | 1.51 | 77.93 | 2.25 | 126.30 |
| Marmande | 4.065 | 0.40 | 2.91 | 62.60 | 4.76 | 22.55 |
| Melillero | 4.624 | 0.27 | 3.26 | 83.77 | 3.65 | 9.53 |
| Mex 89 | 2.328 | 0.13 | 2.13 | 28.40 | 2.62 | 9.92 |
| Moneymaker | 4.077 | 0.47 | 3.24 | 74.79 | 3.69 | 25.36 |
| Monita | 4.286 | 0.87 | 2.42 | 86.53 | 3.94 | 0.47 |
| Moruno | 4.471 | 0.67 | 4.29 | 95.47 | 4.76 | 24.00 |
| PE55 | 3.981 | 0.73 | 3.14 | 64.57 | 4.66 | 47.89 |
| De Penjar | 3.718 | 0.47 | 2.96 | 52.69 | 4.03 | 7.83 |
| Periana | 2.12 | 0.00 | 1.90 | 79.80 | 2.11 | 33.01 |
| PI134418 | 1.437 | 0.13 | 0.93 | 5.71 | 1.71 | 42.84 |
| San Marzano | 4.141 | 0.60 | 3.27 | 75.93 | 4.96 | 31.24 |
| T0 93715 | 4.589 | 0.80 | 3.95 | 68.60 | 4.43 | 47.94 |

|  |  |  |  |  |
| --- | --- | --- | --- | --- |
| **Tomato variety** | **Plant biomass (aphid treatment) (g)** | **Aphid number** | **Plant biomass (nematode treatment) (g)** | **Nematode number** |
| H. de Toro | 3.66 | 70.64 | 4.96 | 13.63 |
| BC5 | 3.13 | 32.86 | 3.42 | 47.14 |
| Edkawi | 2.56 | 66.83 | 4.01 | 79.41 |
| Flor Baladre | 3.09 | 67.60 | 4.10 | 27.21 |
| Kalohi | 3.30 | 81.80 | 4.21 | 21.23 |
| LA1589 | 1.51 | 77.93 | 2.25 | 126.30 |
| Marmande | 2.91 | 62.60 | 4.76 | 22.55 |
| Melillero | 3.26 | 83.77 | 3.65 | 9.53 |
| Mex 89 | 2.13 | 28.40 | 2.62 | 9.92 |
| Moneymaker | 3.24 | 74.79 | 3.69 | 25.36 |
| Monita | 2.42 | 86.53 | 3.94 | 0.47 |
| Moruno | 4.29 | 95.47 | 4.76 | 24.00 |
| PE55 | 3.14 | 64.57 | 4.66 | 47.89 |
| De Penjar | 2.96 | 52.69 | 4.03 | 7.83 |
| Periana | 1.90 | 79.80 | 2.11 | 33.01 |
| PI134418 | 0.93 | 5.71 | 1.71 | 42.84 |
| San Marzano | 3.27 | 75.93 | 4.96 | 31.24 |
| T0 93715 | 3.95 | 68.60 | 4.43 | 47.94 |

**
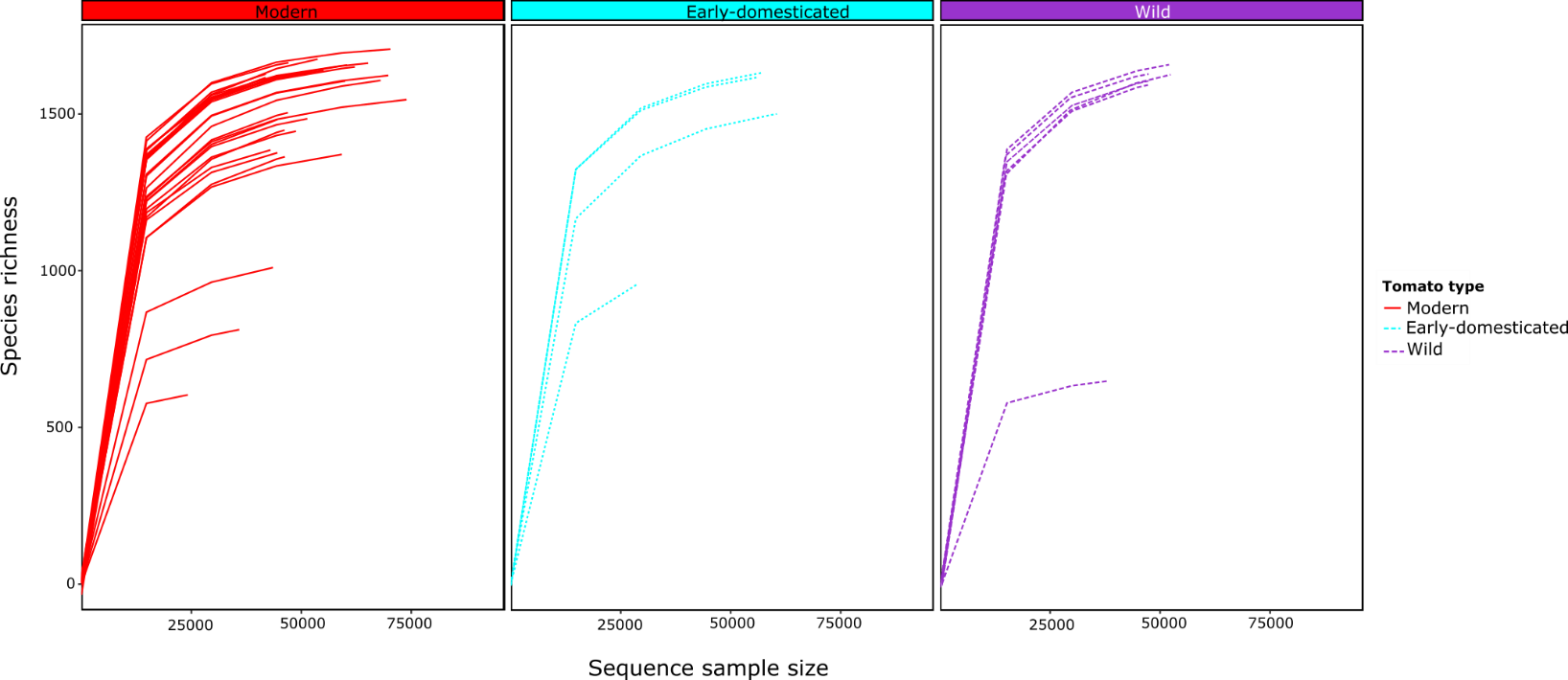
**

Figure S1: Rarefaction curves of 18 varieties of tomato (*Solanum lycopersicum* Mill.. *S. habrochaites* and *S. pimpinellifolium*). Tomato varieties were classified into wild (purple). early-domesticated (light blue) and modern (red).

**Table S2.** Pearson correlation test of bacterial diversity indexes and plant traits and soil variables. R **coefficients** are shown. Asterisks indicate significance: * p < 0.05; ** p < 0.01. For details in plant traits see Table S1.

| **Variable type** | **Variable** | **S** | **Simpson** | **Shannon** |
| --- | --- | --- | --- | --- |
| Plant traits | Biomass (Field exp) | 0.06 | -0.017 | 0.007 |
|  | Tomato Fruit Weight | 0.196 | 0.281 | 0.191 |
|  | Tomato Fruit Number | 0.164 | 0.212 | 0.234 |
|  | Plant State | 0.095 | 0.068 | 0.114 |
|  | Frequency ToCV | 0.034 | 0.024 | 0.023 |
|  | Frequency TYLCV | **0.637 **** | **0.62 **** | **0.685 **** |
|  | Biomass (Ferrero et al (2019)) | 0.02 | 0.099 | 0.083 |
|  | Spodoptera survival | **0.524 *** | **0.604 **** | **0.631 **** |
|  | Aphid Biomass | 0.013 | 0.01 | 0.032 |
|  | Aphid number | -0.211 | -0.185 | -0.21 |
|  | Nematode_biomass | 0.107 | 0.129 | 0.153 |
|  | Nematode number | -0.047 | -0.128 | -0.067 |
| Soil variables | Nitrogen | -0.226 | -0.006 | -0.188 |
|  | Carbon | -0.335 | -0.111 | -0.291 |
|  | C:N ratio | -0.397 | -0.199 | -0.353 |
|  | Al | -0.274 | -0.31 | -0.287 |
|  | As | 0.169 | 0.119 | 0.231 |
|  | Ca | -0.214 | -0.175 | -0.261 |
|  | Cd | 0.399 | 0.276 | 0.409 |
|  | Co | 0.078 | -0.01 | 0.099 |
|  | Cr | -0.229 | -0.275 | -0.246 |
|  | Cu | 0.225 | 0.26 | 0.248 |
|  | Fe | 0.286 | 0.187 | 0.304 |
|  | K | -0.344 | -0.365 | -0.354 |
|  | Li | 0.336 | 0.185 | 0.291 |
|  | Mg | -0.062 | -0.112 | -0.148 |
|  | Mn | 0.205 | 0.127 | 0.231 |
|  | Na | -0.351 | -0.37 | -0.373 |
|  | Ni | 0.351 | 0.238 | 0.361 |
|  | P | -0.379 | -0.365 | -0.315 |
|  | Pb | -0.068 | -0.125 | -0.021 |
|  | S | -0.307 | -0.178 | -0.328 |
|  | Si | **-0.496 *** | **-0.513 *** | **-0.50 *** |
|  | Sr | **-0.479 *** | -0.343 | **-0.486 *** |
|  | Ti | -0.358 | -0.378 | -0.389 |
|  | V | -0.243 | -0.29 | -0.262 |
|  | Zn | 0.252 | 0.14 | 0.208 |

**Table S3.** Stepwise model selection of redundancy analyses for plant traits (fruit and resistance) and soil nutrient variables. Asterisks indicate significance: .p < 0.1; * p < 0.05; ** p < 0.01. For details in plant traits see Table S1.

| **Dataset** | **Variable type** | **Variable** | **Df** | **AIC** | **F** | **p** |
| --- | --- | --- | --- | --- | --- | --- |
| Whole dataset | Resistance traits | Frequency TYLCV | 1 | -37.574 | 22.245 | **0.005**** |
|  | Soil variables | Si | 1 | -37.616 | 17.284 | **0.010**** |
|  |  | Ni | 1 | -37.488 | 18.409 | **0.005**** |
|  |  | CN ratio | 1 | -37.167 | 21.262 | **0.005**** |
| Excluding wild var. | Resistance traits | Frequency TYLCV | 1 | -31.06 | 2.269 | **0.005**** |
|  | Soil variables | As | 1 | -31.510 | 18.147 | **0.020*** |
|  |  | Carbon | 1 | -30.544 | 26.009 | **0.005**** |
|  |  | CN ratio | 1 | -30.498 | 26.396 | **0.005**** |
|  |  | S | 1 | -31.905 | 15.075 | 0.080 . |

**Table S4.** Variation partitioning of bacterial OTU community composition in plant traits (fruit and resistance). tomato phylogeny and soil variables. Either considering the whole dataset or only domesticated tomato varieties. Asterisk indicates significant p values: . p < 0.1; * p < 0.05. For details in plant traits see Table S1.

| **Dataset** | **Partition** | **Df** | **R^2^** |
| --- | --- | --- | --- |
| Whole dataset | Resistance traits | 1 | 0.043 . |
|  | Phylogeny | 4 | 0.054 |
|  | Soil | 2 | **0.094 *** |
|  | Resistance × Phylogeny | 0 | 0 |
|  | Resistance × Soil | 0 | 0 |
|  | Phylogeny × Soil | 0 | 0.053 |
|  | Resist. × Phylo × Soil | 0 | 0 |
|  | All | 0 | 0 |
|  | Residuals | 0 | 0.757 |
| Excluding wild var. | Resistance traits | 1 | **0.097 *** |
|  | Phylogeny | 1 | 0.007 |
|  | Soil | 3 | **0.074 *** |
|  | Resistance × Phylogeny | 0 | 0 |
|  | Resistance × Soil | 0 | 0.050 |
|  | Phylogeny × Soil | 0 | 0 |
|  | Resist. × Phylo × Soil | 0 | 0 |
|  | All | 0 | 0 |
|  | Residuals | 0 | 0.772 |
