# Supplementary material for: Resistance and Not Plant Fruit Traits Determine Root-Associated Bacterial Community Composition along a Domestication Gradient in Tomato
Source: Plants (Basel). 2021 Dec 23;11(1):43. doi: 10.3390/plants11010043 (PMC8747438; doi:10.3390/plants11010043)
Supplement: Supplementary file 1 [file plants-11-00043-s001.zip › SupplementaryTables.pdf]

Supplementary Material

Table S1. Experimental variables included in the multivariate analysis. A) Domestication degree per tomato variety. B) Data from the field experiment: plant traits. C) Data from field experiment: soil characterization. D) Data from Ferrero et al. 2019: plant traits.

Domestication degree

Tomato variety	Species	Domestication degree
H. de Toro	*Solanum lycopersicum*	Modern
BC5	*Solanum lycopersicum*	Modern
Edkawi	*Solanum lycopersicum*	Modern
Flor Baladre	*Solanum lycopersicum*	Modern
Kalohi	*Solanum lycopersicum*	Modern
LA1589	*Solanum pimpinellifolium*	Wild
Marmande	*Solanum lycopersicum*	Modern
Melillero	*Solanum lycopersicum*	Modern
Mex 89	*Solanum lycopersicum* var. *cerasiforme*	Early-domesticated
Moneymaker	*Solanum lycopersicum*	Modern
Monita	*Solanum lycopersicum*	Modern
Moruno	*Solanum lycopersicum*	Modern
PE55	*Solanum lycopersicum*	Early-domesticated
De Penjar	*Solanum lycopersicum*	Modern
Periana	*Solanum lycopersicum var. cerasiforme*	Modern
PI134418	*Solanum habrochaites*	Wild
San Marzano	*Solanum lycopersicum*	Modern

T0 93715	*Solanum pimpinellifolium*	Wild

B) Plant traits field experiment. Plant state: Plant symptom characterization; Frequency ToCV: Percentage of tomato replicates with chlorosis virus detected with tissue-blot hybridization; Frequency TYLCV: Percentage of tomato replicates with yellow leaf curl virus detected with tissue-blot hybridization.

Tomato variety	Total plant biomass (Kg)	Tomato fruit weight (Kg)	Tomato fruit number	State	Frequency ToCV	Frequency TYLCV
H. de Toro	0.41	99.2	0.5	3.00	1.00	0.8
BC5	0.57	207.91	5.7	4.3	0.20	0.3
Edkawi	0.325	107.05	0.875	2.75	1.00	0.7
Flor Baladre	0.33	225.04	0.33	2.22	1.00	0.89
Kalohi	0.39	165.33	1.1	2.2	1.00	0.6
LA1589	0.526	51.06	61.3	6.6	0.80	0.8
Marmande	0.33	412.95	3.5	2.33	1.00	0.5
Melillero	0.52	201.94	1.8	2.7	0.9	0.4
Mex 89	0.76	85.19	65.78	5.78	0.9	0.8
Moneymaker	0.363	403.82	4.2	3.00	1.00	0.7
Monita	0.29	98.24	2.09	2.45	1.00	0.7
Moruno	0.24	137.82	4.00	2.29	1.00	0.25
PE55	0.28	46.00	5.17	3.00	1.00	0.625
De Penjar	0.6725	282.56	12.5	3.9	1.00	0.4
Periana	0.5	69.34	0.3	2.7	0.9	0.1
PI134418	0.39	21.12	17.89	7.44	0.67	0.5
San Marzano	0.63	110.17	2.11	2.44	1.00	0.5
T0 93715	0.63	7.88	15.2	7.4	0.4	0.6

C) Field experiment Chemical composition of soil (ppm (mg/Kg))

Tomato variety	Nitrogen (%)	Carbon (%)	C:N ratio	Al	As	Ca	Cd	Co	Cr	Cu	Fe	K	Li
H. de Toro	0.29	5.48	19.07	32272.45	30.64	9512.67	2.29	21.63	47.01	39.16	32775.74	7891.33	29.24
BC5	0.30	5.77	19.25	29903.35	26.45	10998.40	2.23	19.42	44.87	40.29	31155.15	7694.72	26.80
Edkawi	0.31	6.03	19.47	30849.13	29.29	9355.71	2.23	21.20	45.56	39.34	33169.75	7519.68	28.77
Flor Baladre	0.25	4.00	16.03	40469.74	30.25	8871.26	2.40	22.48	54.91	40.06	35588.47	9655.52	33.43
Kalohi	0.31	5.25	17.18	34563.67	27.25	11063.83	2.07	19.38	48.22	38.63	31031.98	8877.17	27.95
LA1589	0.30	5.79	19.14	44769.84	32.79	7804.21	2.22	21.64	57.71	40.92	33620.68	11887.42	31.11
Marmande	0.36	9.51	26.35	47279.17	33.44	10332.83	2.09	22.12	59.18	38.17	31873.98	12308.45	28.36
Melillero	0.27	5.05	19.05	48191.47	32.23	8737.64	2.27	21.26	61.57	38.24	34490.29	12655.27	31.47
Mex 89	0.24	3.57	14.78	36127.94	35.02	7748.17	2.40	22.77	50.77	40.46	35408.39	8917.46	30.71
Moneymaker	0.32	6.79	21.11	39290.18	31.92	8788.23	2.22	20.71	52.42	41.88	33572.50	9825.97	28.13
Monita	0.35	7.83	22.27	36863.42	27.85	9099.69	2.15	20.85	50.65	39.99	32321.24	9701.17	27.78
Moruno	0.31	6.69	21.30	33901.28	28.67	10937.73	2.13	20.73	47.21	38.34	31601.04	8521.75	26.27
PE55	0.21	3.42	16.68	44845.01	37.77	8121.07	2.40	23.68	57.48	39.02	35385.81	11384.10	30.59
De Penjar	0.26	4.19	15.89	43683.51	31.67	8550.81	2.29	22.27	56.89	38.22	34266.37	11362.56	30.16
Periana	0.32	6.75	21.14	46608.99	23.73	11773.04	2.03	20.42	58.46	38.14	30811.28	12562.43	28.25
PI134418	0.29	5.16	18.05	52079.30	35.37	8814.62	2.42	24.27	64.50	40.21	35617.88	13259.06	32.10
San Marzano	0.29	5.32	18.33	48274.30	25.99	7567.39	2.14	22.05	59.35	34.75	33049.06	12385.03	28.82
T0 93715	0.33	5.44	16.44	34162.53	33.24	7739.21	2.26	21.82	48.03	38.65	33498.50	8794.56	27.40

**Tomato variety**	**Mg**	**Mn**	**Na**	**Ni**	**P**	**Pb**	**S**	**Si**	**Sr**	**Ti**	**V**	**Zn**
H. de Toro	7987.08	846.29	0.04	45.93	707.46	22.47	411.73	2877.92	47.73	1148.59	59.06	125.51
BC5	8344.68	771.62	0.03	40.74	715.53	21.64	489.64	2728.97	57.07	1128.88	56.16	131.67
Edkawi	7550.65	875.68	0.03	43.74	818.07	22.09	470.31	2652.96	58.89	1077.34	56.82	106.75
Flor Baladre	8372.00	863.50	0.04	48.04	767.09	23.16	373.89	3398.72	52.20	1318.87	69.95	109.48
Kalohi	8465.32	859.65	0.04	41.15	832.00	21.53	481.18	3204.44	54.61	1212.39	60.98	99.80
LA1589	7584.11	888.58	0.05	45.09	968.39	23.32	409.23	4196.16	50.89	1375.55	73.44	84.85
Marmande	8032.74	925.72	0.06	43.99	992.85	23.92	487.64	4799.41	61.69	1490.84	77.27	84.34
Melillero	8438.64	807.50	0.05	45.56	887.12	23.33	388.67	4237.66	51.75	1534.15	79.85	83.94
Mex 89	7884.02	980.78	0.03	48.31	1020.34	24.51	341.58	3054.83	42.80	1191.39	64.45	97.92
Moneymaker	8131.44	727.50	0.04	43.16	822.85	23.24	406.64	3350.30	54.84	1232.17	66.60	91.76
Monita	7800.04	793.15	0.04	43.02	1072.14	22.11	471.51	3359.55	53.64	1332.99	63.90	85.51
Moruno	8424.78	882.49	0.04	42.95	933.49	23.01	422.84	3230.94	56.79	1149.81	59.14	96.92
PE55	7764.41	1022.06	0.05	48.33	1236.94	25.20	314.81	3843.06	47.52	1304.76	73.95	100.50
De Penjar	8187.77	874.02	0.05	45.86	1064.76	24.39	390.81	3542.49	50.39	1380.06	73.00	103.77
Periana	8992.66	729.31	0.06	40.29	937.59	22.04	547.08	4490.26	65.13	1555.12	76.24	96.26
PI134418	8677.31	968.29	0.06	49.26	1174.52	24.65	414.23	3914.28	47.82	1479.69	84.41	93.77
San Marzano	7979.41	1177.24	0.06	49.03	992.56	21.64	347.10	4147.62	49.36	1422.21	79.08	84.83
T0 93715	7232.60	955.40	0.03	45.16	1129.40	24.36	404.48	4089.26	47.52	1175.80	60.73	107.31

D) Ferrero et al. (2019) resistance traits. Averages per tomato variety. Total plant biomass (g): Total plant biomass (dry weight) of control plants (no pest attack); Spodoptera exigua survival: Mean increase in weight per day; Plant biomass (aphid treatment) (g): Plant biomass (dry weight) under aphid pressure; Aphid number: Number of aphids in the plant at the end of experiment; Plant biomass (nematode treatment)(g): Plant biomass (dry weight) under nematode pressure; Nematode number: Number of root knots/ mg root.

Tomato variety	Total plant biomass (g)	Spodoptera exigua survival	Plant biomass (aphid treatment) (g)	Aphid number	Plant biomass (nematode treatment) (g)	Nematode number
H. de Toro	3.997	0.47	3.66	70.64	4.96	13.63
BC5	3.708	0.33	3.13	32.86	3.42	47.14
Edkawi	4.746	0.60	2.56	66.83	4.01	79.41
Flor Baladre	3.818	0.60	3.09	67.60	4.10	27.21
Kalohi	3.901	0.33	3.30	81.80	4.21	21.23
LA1589	2.815	0.27	1.51	77.93	2.25	126.30
Marmande	4.065	0.40	2.91	62.60	4.76	22.55
Melillero	4.624	0.27	3.26	83.77	3.65	9.53
Mex 89	2.328	0.13	2.13	28.40	2.62	9.92
Moneymaker	4.077	0.47	3.24	74.79	3.69	25.36
Monita	4.286	0.87	2.42	86.53	3.94	0.47
Moruno	4.471	0.67	4.29	95.47	4.76	24.00
PE55	3.981	0.73	3.14	64.57	4.66	47.89
De Penjar	3.718	0.47	2.96	52.69	4.03	7.83
Periana	2.12	0.00	1.90	79.80	2.11	33.01
PI134418	1.437	0.13	0.93	5.71	1.71	42.84
San Marzano	4.141	0.60	3.27	75.93	4.96	31.24
T0 93715	4.589	0.80	3.95	68.60	4.43	47.94

Tomato variety	Plant biomass (aphid treatment) (g)	Aphid number	Plant biomass (nematode treatment) (g)	Nematode number
H. de Toro	3.66	70.64	4.96	13.63
BC5	3.13	32.86	3.42	47.14
Edkawi	2.56	66.83	4.01	79.41
Flor Baladre	3.09	67.60	4.10	27.21
Kalohi	3.30	81.80	4.21	21.23
LA1589	1.51	77.93	2.25	126.30
Marmande	2.91	62.60	4.76	22.55
Melillero	3.26	83.77	3.65	9.53
Mex 89	2.13	28.40	2.62	9.92
Moneymaker	3.24	74.79	3.69	25.36
Monita	2.42	86.53	3.94	0.47
Moruno	4.29	95.47	4.76	24.00
PE55	3.14	64.57	4.66	47.89
De Penjar	2.96	52.69	4.03	7.83
Periana	1.90	79.80	2.11	33.01
PI134418	0.93	5.71	1.71	42.84
San Marzano	3.27	75.93	4.96	31.24
T0 93715	3.95	68.60	4.43	47.94

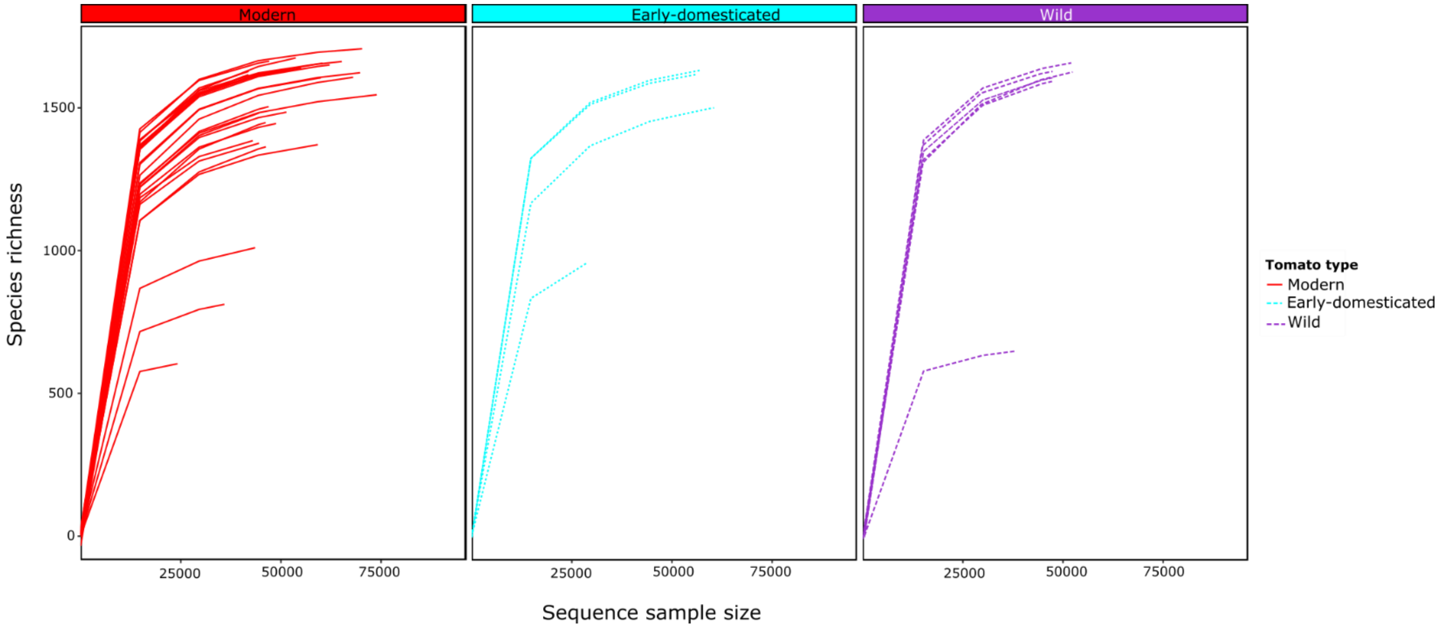

Figure S1: Rarefaction curves of 18 varieties of tomato (*Solanum lycopersicum* Mill.. *S. habrochaites* and *S. pimpinellifolium*). Tomato varieties were classified into wild (purple). early-domesticated (light blue) and modern (red).

Table S2. Pearson correlation test of bacterial diversity indexes and plant traits and soil variables. R **coefficients** are shown. Asterisks indicate significance: * $p < 0.05$; ** $p < 0.01$. For details in plant traits see Table S1.

Variable type	Variable	S	Simpson	Shannon
Plant traits	Biomass (Field exp)	0.06	-0.017	0.007
	Tomato Fruit Weight	0.196	0.281	0.191
	Tomato Fruit Number	0.164	0.212	0.234
	Plant State	0.095	0.068	0.114
	Frequency ToCV	0.034	0.024	0.023
	Frequency TYLCV	**0.637 ****	**0.62 ****	**0.685 ****
	Biomass (Ferrero et al (2019))	0.02	0.099	0.083
	Spodoptera survival	**0.524 ***	**0.604 ****	**0.631 ****
	Aphid Biomass	0.013	0.01	0.032
	Aphid number	-0.211	-0.185	-0.21

	Nematode_biomass	0.107	0.129	0.153
	Nematode number	-0.047	-0.128	-0.067
Soil variables	Nitrogen	-0.226	-0.006	-0.188
	Carbon	-0.335	-0.111	-0.291
	C:N ratio	-0.397	-0.199	-0.353
	Al	-0.274	-0.31	-0.287
	As	0.169	0.119	0.231
	Ca	-0.214	-0.175	-0.261
	Cd	0.399	0.276	0.409
	Co	0.078	-0.01	0.099
	Cr	-0.229	-0.275	-0.246
	Cu	0.225	0.26	0.248
	Fe	0.286	0.187	0.304

K	-0.344	-0.365	-0.354
Li	0.336	0.185	0.291
Mg	-0.062	-0.112	-0.148
Mn	0.205	0.127	0.231
Na	-0.351	-0.37	-0.373
Ni	0.351	0.238	0.361
P	-0.379	-0.365	-0.315
Pb	-0.068	-0.125	-0.021
S	-0.307	-0.178	-0.328
Si	**-0.496 ***	**-0.513 ***	**-0.50 ***
Sr	**-0.479 ***	-0.343	**-0.486 ***
Ti	-0.358	-0.378	-0.389
V	-0.243	-0.29	-0.262

Zn	0.252	0.14	0.208

Table S3. Stepwise model selection of redundancy analyses for plant traits (fruit and resistance) and soil nutrient variables. Asterisks indicate significance: .p < 0.1; * p < 0.05; ** p < 0.01. For details in plant traits see Table S1.

**Dataset**	**Variable type**	**Variable**	**Df**	**AIC**	**F**	**p**
Whole dataset	Resistance traits	Frequency TYLCV	1	-37.574	22.245	**0.005***
	Soil variables	Si	1	-37.616	17.284	**0.010***
		Ni	1	-37.488	18.409	**0.005***
		CN ratio	1	-37.167	21.262	**0.005***
Excluding wild var.	Resistance traits	Frequency TYLCV	1	-31.06	2.269	**0.005***
	Soil variables	As	1	-31.510	18.147	**0.020***
		Carbon	1	-30.544	26.009	**0.005***
		CN ratio	1	-30.498	26.396	**0.005***
		S	1	-31.905	15.075	0.080 .

Table S4. Variation partitioning of bacterial OTU community composition in plant traits (fruit and resistance). tomato phylogeny and soil variables. Either considering the whole dataset or only domesticated tomato varieties. Asterisk indicates significant p values: . $p < 0.1$; * $p < 0.05$. For details in plant traits see Table S1.

Dataset	Partition	Df	R^2
Whole dataset	Resistance traits	1	0.043 .
	Phylogeny	4	0.054
	Soil	2	**0.094 ***
	Resistance × Phylogeny	0	0
	Resistance × Soil	0	0
	Phylogeny × Soil	0	0.053
	Resist. × Phylo × Soil	0	0
	All	0	0
	Residuals	0	0.757
Excluding wild var.	Resistance traits	1	**0.097 ***

Phylogeny	1	0.007
Soil	3	**0.074 ***
Resistance × Phylogeny	0	0
Resistance × Soil	0	0.050
Phylogeny × Soil	0	0
Resist. × Phylo × Soil	0	0
All	0	0
Residuals	0	0.772
