# Supplementary figures and images for: Resistance and Not Plant Fruit Traits Determine Root-Associated Bacterial Community Composition along a Domestication Gradient in Tomato
Source: Plants (Basel). 2021 Dec 23;11(1):43. doi: 10.3390/plants11010043 (PMC8747438; doi:10.3390/plants11010043)

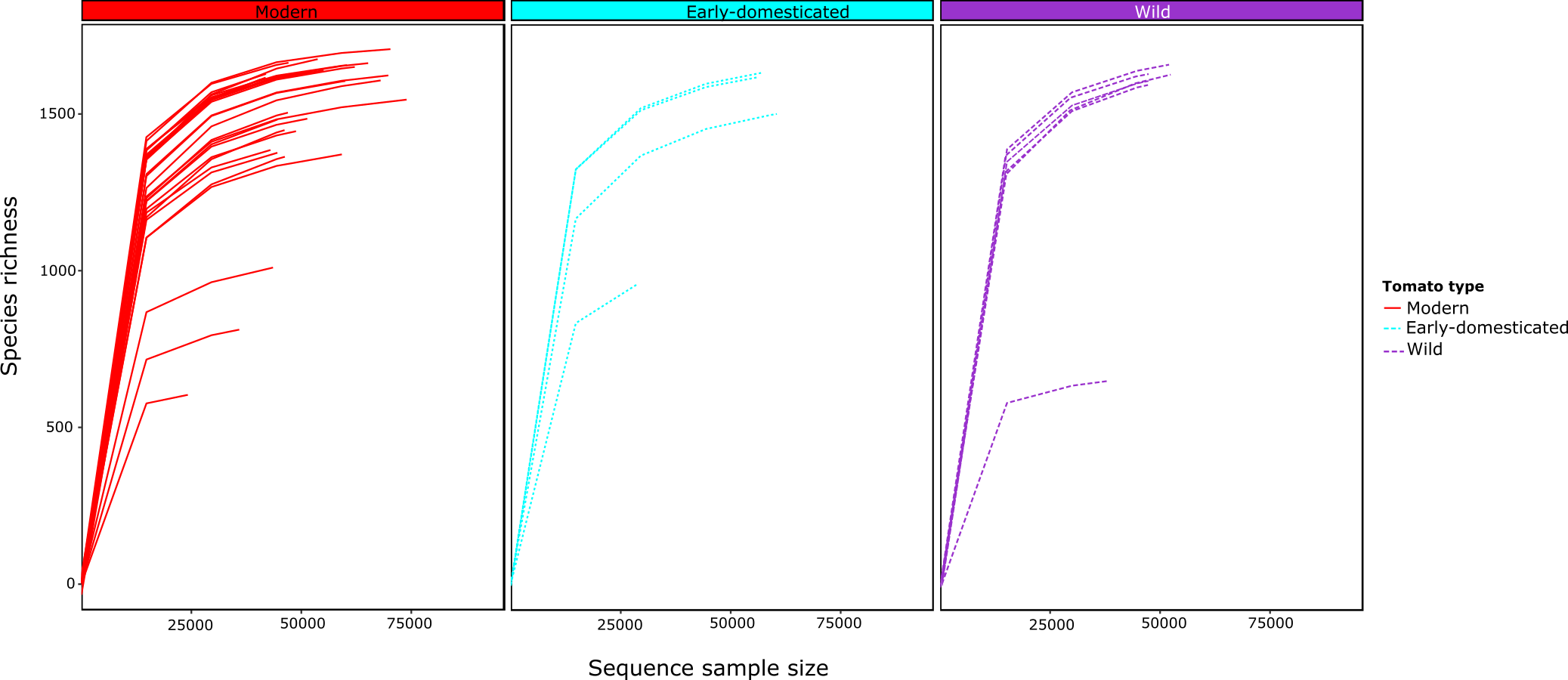

Supplement: Supplementary file 1 [file plants-11-00043-s001.zip › Figure S1.png]
